# Supplementary material for: Differentially Expressed Genes and Signalling Pathways Regulated by High Selenium Involved in Antioxidant and Immune Functions of Goats Based on Transcriptome Sequencing
Source: Int J Mol Sci. 2023 Jan 6;24(2):1124. doi: 10.3390/ijms24021124 (PMC9864924; doi:10.3390/ijms24021124)
Supplement: Supplementary file 1 [file ijms-24-01124-s001.zip › Table S1. Ingredients and nutrient composition of basal diets (DM basis).pdf]

Table S1. Ingredients and nutrient composition of basal diets (DM basis)

**Ingredients (% of fed basis)**	**Content**
Peanut vines	50.00
White distiller's grains	10.00
Soybean residues	10.00
Green hay	9.30
Corn	16.00
Soybean meal	3.00
Premix	1.00
Salt	0.50
Limestone	0.20
Total	100.00
Chemical composition (% of DM)	
Dry matter, %	90.15
Crude protein	11.95
Neutral detergent fiber	42.74
Acid detergent fiber	28.61
Ether extract	2.35
Ash	8.72

Premix contained per kilogram feed: vitamin A was 4000000 IU, vitamin D was 600000 IU, vitamin E was 25000 mg, DL-methionine was 7000 mg, L-lysine was 5000 mg, Cu was 1300 mg, Fe was 1000 mg, Zn was 1575 mg, and Mn was 595 mg.
