# Supplementary material for: Differentially Expressed Genes and Signalling Pathways Regulated by High Selenium Involved in Antioxidant and Immune Functions of Goats Based on Transcriptome Sequencing
Source: Int J Mol Sci. 2023 Jan 6;24(2):1124. doi: 10.3390/ijms24021124 (PMC9864924; doi:10.3390/ijms24021124)
Supplement: Supplementary file 1 [file ijms-24-01124-s001.zip › Table S8. Information for candidate genes used for real-time PCR in this study.pdf]

Table S8. Information for candidate genes used for real-time PCR in this study.

Gene	Primer sequences (5′ to 3′)	Accession number	Product size, nt	Annealing temperature, ºC
SEPW1	(F) TGGACACGGAGAGCAAGTTT	XM_005709381.3	94	60
	(R) CGGTTACTGGTCTCTGCCTT			
GPX1	(F) GCATCGCTCTGAGGCACAAC	XM_005695962.3	134	60
	(R) TCGTTCTTGGCATTTTCCTGA			
GSTA1	(F) CCGCCAAACACAACCTCTAC	NM_001314321.1	79	60
	(R) AAATCTGCCACACCCTCTGA			
TNF	(F) TTCAGACACTCAGGTCATCTTCTCA	NM_001286442.1	118	60
	(R) TTGAGGGCATTGGCATACGA			
TNFSF10	(F) ATGGCTTGGGTTGCTTCTTG	XM_005675308.3	85	60
	(R) AGTTCCCAGCAGGGATTGAT			
TNFSF8	(F) GGTGATCCAGTTCCCAGGTT	XM_005684351.3	154	60
	(R) TTGTGCTCCAGACTCACACA			
TNFSF13B	(F) GGACGAACTGAGTCTGGTGA	NM_001285702.1	122	60
	(R) CCAGTTGGAGTTCGTCTCCT			
GAPDH	(F) GCCCTCTCAAGGGCATTCTA	XM_005680968.3	81	60
	(R) AGGTAGAAGAGTGAGTGTCGC			

SEPW1, selenoprotein W, 1; *GPX1*, glutathione peroxidase 1; *GSTA1*, glutathione S-transferase A1; *TNF*, tumor necrosis factor; *TNFSF10*, tumor necrosis factor superfamily member 10; *TNFSF8*, tumor necrosis factor superfamily member 8; *TNFSF13B*, tumor necrosis factor superfamily member 13b; *GAPDH*, glyceraldehyde-3-phosphate dehydrogenase.
